# PUBLIC REPORTING OF NH ANTIPSYCHOTIC USE: CHANGES IN THE REPORTING OF EXCLUSIONARY DIAGNOSES?

**DOI:** 10.1093/geroni/igz038.2838

**Published:** 2019-11-08

**Authors:** Judith A Lucas, John R Bowblis, Mary Lynn Davis-Ajami, Kosali Simon, Miyeon Song

**Affiliations:** 1 Seton Hall University, South Orange, New Jersey, United States; 2 Miami University, Oxford, Ohio, United States; 3 Indiana University, Bloomington, Indiana, United States; 4 Texas A&M University, College Station, Texas, United States

## Abstract

Antipsychotics (APMs) are commonly used off-label to control behavioral symptoms of dementia in nursing home (NH) residents despite FDA warnings. As part of the Centers for Medicare and Medicaid Services’ (CMS) partnership to reduce the use of APMs in long-stay NH residents with dementia, CMS began publicly reporting an APM quality measure on the NH Compare website in July of 2012. This measure proxies for potentially “inappropriate” use by excluding residents with diagnoses in which APMs may be appropriate. While APM use as declined, some question if the quality measure creates an incentive to change documentation of exclusionary diagnoses to show improvements in the publicly reported APM quality measure. These exclusionary diagnoses include: Huntington’s disease, Tourette syndrome, and schizophrenia. Using Minimum Data Set (MDS) assessments from long-stay NH residents in freestanding NHs (2011-2015), we utilized a difference-in-differences framework to compared the proportion of assessments with exclusionary diagnoses in NHs that were required to report the APM quality measure (treatment) to NHs that were exempt from reporting the measure (control). We also stratified the results by grouping residents under and over 65 year of age. We find no change in exclusionary diagnoses immediately following the implementation of public reporting. However, starting in 2014, the use of exclusionary diagnoses increased in the treatment group, especially among residents aged 65+. While APM use declined for all groups over the study period, our results indicate that the reduction in APM use may not be as large as expected, especially after 2014.

